# Methotrexate persistence and adverse drug reactions in patients with juvenile idiopathic arthritis

**DOI:** 10.1093/rheumatology/kez048

**Published:** 2019-03-08

**Authors:** Lianne Kearsley-Fleet, Laura Vicente González, Douglas Steinke, Rebecca Davies, Diederik De Cock, Eileen Baildam, Michael W Beresford, Helen E Foster, Taunton R Southwood, Wendy Thomson, Kimme L Hyrich, Biologics for Children with Rheumatic Diseases (BCRD) Study and the British Society for Paediatric and Adolescent Rheumatology Etanercept Cohort Study (BSPAR-ETN)

**Affiliations:** 1 Arthritis Research UK Centre for Epidemiology, Manchester Academic Health Science Centre, University of Manchester, Manchester, UK; 2 Division of Pharmacy and Optometry, School of Health Sciences, University of Manchester, Manchester, UK; 3 Clinical Academic Department of Paediatric Rheumatology, Alder Hey Children’s NHS Foundation Trust, Liverpool, UK; 4 Institute of Translational Medicine (Child Health), University of Liverpool, Liverpool, UK; 5 Musculoskeletal Research Group, Institute of Cellular Medicine, Newcastle University, Newcastle upon Tyne, UK; 6 Paediatric Rheumatology, Great North Children’s Hospital, Newcastle upon Tyne, UK; 7 Institute of Child Health, University of Birmingham and Birmingham Children’s Hospital, Birmingham, UK; 8 Arthritis Research UK Centre for Genetics and Genomics, Centre for Musculoskeletal Research, Faculty of Biologic, Medicine and Health, University of Manchester, Manchester, UK; 9 National Institute of Health Research Manchester Biomedical Research Centre, Manchester Academic Health Science Centre, Manchester University NHS Foundation Trust, Manchester, UK

**Keywords:** DMARDs, epidemiology, juvenile idiopathic arthritis, outcome measures, statistics

## Abstract

**Objectives:**

This analysis aims to calculate MTX monotherapy persistence and describe the occurrence of and factors associated with the occurrence of adverse drug reactions (ADRs) with MTX.

**Methods:**

Patients with JIA starting MTX monotherapy from two UK studies were included. Patient characteristics, treatment details and ADR occurrence were collected at treatment start, 6 months, 1 year and annually. The following groups of ADRs were included: gastrointestinal, elevated liver enzymes, leukopenia, drug hypersensitivity, rash, needle phobia and any events leading to permanent MTX discontinuation. Treatment exposure was calculated from MTX start until MTX monotherapy cessation, last follow-up or 31 December 2017 (cut-off), whichever came first. Survival analysis assessed the time on MTX monotherapy and the time to the first ADR on MTX monotherapy within 2 years. Multivariable logistic regression assessed characteristics associated with any ADR and gastrointestinal ADRs.

**Results:**

A total of 577 patients started MTX. At 2 years, 310 (54%) were no longer on MTX monotherapy. Reasons included ineffectiveness (60%; 161/185 started a biologic), adverse event (25%), remission (8%) and patient/family decision (3%). Over this time, 212 (37%) patients experienced one or more ADR; commonly gastrointestinal (68%) or elevated liver enzymes (26%). Lower physician global assessment and older age predicted any ADR and gastrointestinal ADR, respectively. Patients with polyarticular RF and JIA had reduced odds of both any ADR and a gastrointestinal ADR.

**Conclusion:**

After 2 years, more than half the patients were no longer on MTX monotherapy, while more than one-third experienced one or more ADR, most commonly gastrointestinal. Research focusing on identifying which children will respond and/or experience ADRs is crucial to inform treatment decisions and management planning.

**Rheumatology key messages**

- Many JIA patients (46%) remain on methotrexate as their sole DMARD at 2 years.
- One-third of JIA patients experienced an adverse drug reaction on methotrexate monotherapy over 2 years.
- Gastrointestinal adverse drug reactions were common in older patients but less likely in polyarticular rheumatoid factor–positive JIA.

## Introduction

JIA is the most common chronic inflammatory rheumatic condition in children [1]. MTX is the current recommended first-line conventional synthetic DMARD [2]. It is an effective treatment [3] with a good safety profile [4]. However, it is estimated up to a third of children do not respond and a further proportion of children are unable to tolerate the medicine [5]. Intolerance and nausea with MTX have been reported repeatedly, ranging up to 73% [6–8]. Factors previously found to be associated with MTX intolerance include prolonged MTX use, s.c. administration, polyarticular JIA and adolescents rather than young adults [6, 8, 9].

Treatment persistence is considered a good surrogate for treatment effectiveness and tolerance [10]. The occurrence of adverse drug reactions (ADRs) can influence persistence with therapies. However, data on how long patients remain on MTX therapy are sparse, ranging from 55% to 93% at 1 year [6, 11–13]. However, very few patients in those cohorts reported starting a biologic therapy, with one excluding these patients completely. Patients who are intolerant or not responding to MTX are now likely to start a biologic, often in addition to rather than instead of MTX [14], making overall persistence with MTX a less reliable estimate of effectiveness and tolerance if the addition of another anti-rheumatic therapy is not also considered.

The UK has two of the largest prospective observational studies of children and young people with JIA receiving MTX therapy [15]. The aims of this analysis were therefore to calculate persistence of MTX as a monotherapy in patients with JIA over the first 2 years following the initiation of therapy, describe reasons for MTX monotherapy treatment discontinuation, quantify and describe the types of ADRs patients experience over the same time period and investigate clinical factors associated with the occurrence of an ADR.

## Methods

### Study cohort

There are two parallel UK prospective, observational cohort studies investigating new biologic therapies for JIA: the British Society for Paediatric and Adolescent Rheumatology Etanercept Cohort Study [16], approved by the West Midlands Research Ethic Committee (initiated in 2004) and the Biologics for Children with Rheumatic Diseases study [17], approved by the North West 7 Research Ethics Committee Greater Manchester Central Ethics Committee (initiated in 2010). In addition to biologic therapies, these registers also recruit cohorts of patients starting MTX. Patients (or parents, if appropriate) provided written informed consent, in accordance with the Declaration of Helsinki. Additional ethical approval was not required to undertake this current analysis.

This analysis was restricted to patients with JIA registered at the point of starting MTX for the first time from 1 January 2010 until 31 December 2015 (to allow ⩾2 years of follow-up). At registration (start of MTX), the physician or clinical research nurse collects patient demographics (age, gender), disease features including ILAR category, disease activity [active joint count, limited joint count, physician global assessment of disease activity (PGA), parent/patient global assessment of well-being, Childhood Health Assessment Questionnaire (CHAQ), pain visual analogue scale, ESR and CRP] and other anti-rheumatic therapies. The 71-joint juvenile arthritis disease activity score (JADAS) was calculated using the core outcome measures [18]. At 6 months, 1 year and then annually, follow-up data were collected on changes in disease activity or anti-rheumatic therapy and the occurrence of any adverse events. All adverse events were coded using the Medical Dictionary for Regulatory Activities [19].

### MTX survival

MTX monotherapy was defined as the time during which a patient was receiving MTX as their sole DMARD for JIA. Time on MTX as monotherapy was calculated from the start date of MTX until the patient stopped MTX for the first time or started an additional DMARD or biologic therapy. Patients still on MTX monotherapy were censored at the last recorded study follow-up date, 31 December 2017 (cut-off date) or death, whichever came first. Patients who stopped therapy temporarily for whatever reason for <90 days were considered to be receiving continuous therapy. MTX drug survival was presented as a Kaplan–Meier curve over the first 2 years of therapy.

### ADRs

ADRs were identified from the reported adverse events based on events that reflected the most common ADRs associated with MTX: nausea, vomiting, elevated liver enzymes, low white blood cell count, injection site reaction, rash, needle phobia and any event that resulted in the permanent cessation of MTX. ADRs were then grouped: gastrointestinal problems (including nausea, vomiting, abdominal pain), elevated liver enzymes, leukopenia, drug hypersensitivity (including injection site reaction), psychological symptoms (including needle phobia, anxiety), rash or other. Events not considered an adverse event (e.g. planned surgery) or not deemed associated directly with MTX (e.g. gastroenteritis, viral rash) were excluded. For each patient, only the first ADR experienced within each category was included, although patients could experience ADRs in multiple categories; this was to restrict overreporting of the same event or ongoing events. For each ADR included, it was noted whether the event led to permanent drug discontinuation. Kaplan–Meier curves were constructed to analyse the time to the first ADR on MTX monotherapy over the first 2 years of therapy. Baseline characteristics associated with experiencing an ADR (yes/no) were assessed using multivariable logistic regression. A sensitivity analysis investigated baseline characteristics associated with experiencing a gastrointestinal ADR only.

Multiple imputation (63 iterations) was used to account for missing data. Imputed values included disease duration and disease activity measures at the start of MTX (active joint count, limited joint count, PGA, parent/patient global assessment of well-being, CHAQ, ESR, CRP and 71-joint JADAS). Stata version 13 (StataCorp, College Station, TX, USA) was used to perform all analyses.

## Results

### Baseline characteristics

A total of 577 patients were included in this analysis; 68% were female, median age at MTX start was 9 years (IQR 4–13) and median disease duration was <1 year (IQR 0–1) (Supplementary Table S1, available at *Rheumatology* online). RF-negative polyarthritis (33%) and persistent oligoarthritis (21%) were the most common ILAR categories. At the start of MTX, 26% received concomitant steroids, the median 71-joint JADAS was 12 (IQR 7–21) and the median CHAQ was 0.9 (IQR 0.3–1.5). The median prescribed dose of MTX was 15 mg/m^2^ (IQR 10–17.5).

### MTX drug survival

**Fig. 1:**
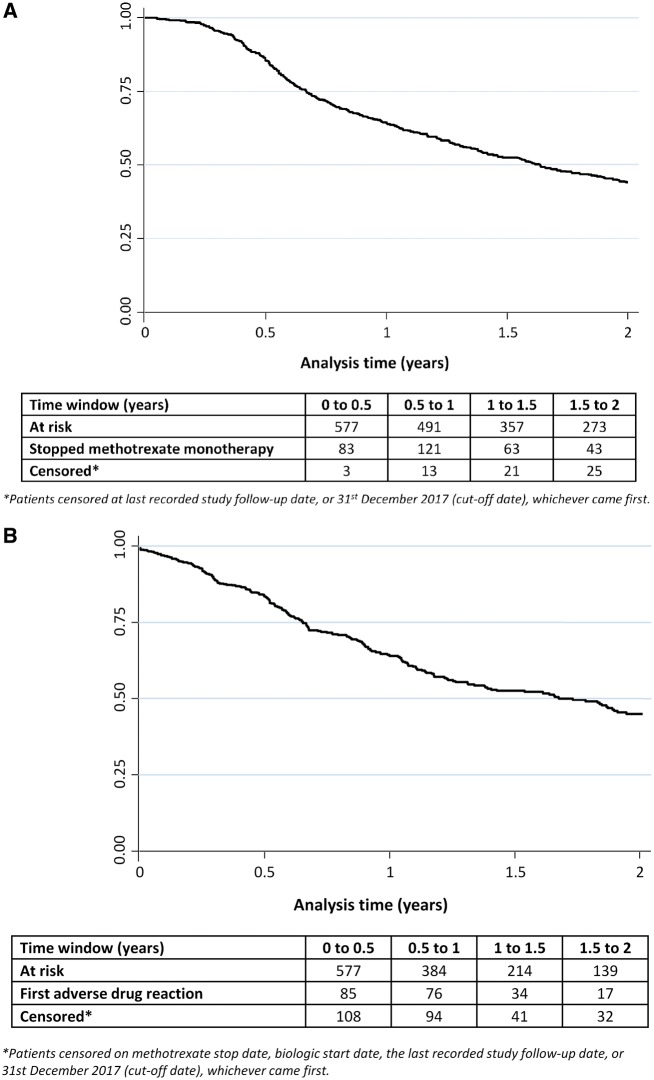
Kaplan–Meier survival graphs of patients with JIA on MTX monotherapy (**A**) Survival analysis in patients with JIA over the first 2 years of MTX monotherapy (*N* = 577). (**B**) Time to first ADR over the first 2 years of MTX monotherapy (*N* = 577).

A total time of 958 person-years on MTX monotherapy was observed with a median monotherapy persistence of 1.1 years (IQR 0.6–2.1). Within 2 years of starting treatment, 310 (54%) patients were no longer receiving MTX as monotherapy (Fig. 1A). Reasons for MTX monotherapy discontinuation reported by the physician included ineffectiveness (60%), of which the majority [161/185 (87%)] added a biologic therapy to their MTX; adverse event (25%); remission (8%) and patient/family decision (3%) (Supplementary Table S2, available at *Rheumatology* online).

### ADRs

During the first 2 years of follow-up, 212 (37%) patients were recorded as experiencing at least one ADR on MTX monotherapy, with the median time to first ADR of 0.6 years (IQR 0.3–1.0) (Fig. 1B). Of the patients with an ADR, 84% had an ADR from one category, 15% had an ADR from two and three patients had an ADR from three or more categories. Most patients with at least one ADR had gastrointestinal problems (68%; mostly nausea and vomiting), followed by elevated liver enzymes (26%), rash (10%), psychological symptoms (3%; mostly anxiety) and drug hypersensitivity (2%) (Supplementary Table S3, available at *Rheumatology* online). Of these ADRs, 16% resulted in permanent discontinuation of MTX therapy.

In the multivariable analysis (Table 1), patients less likely to experience an ADR were those with polyarticular RF-positive JIA compared with polyarticular RF-negative JIA and those with a higher PGA at the start of MTX therapy. When investigating gastrointestinal ADRs only, patients with polyarticular RF-positive JIA were again less likely to experience a gastrointestinal ADR, although for every year increase in age, patients had a 10% increased odds of experiencing a gastrointestinal ADR.

**Table 1:** Multivariable association between baseline characteristics and occurrence of an ADR

Characteristics	OR (95% CI) for experiencing any ADR	OR (95% CI) for experiencing a gastrointestinal ADR
Female (*vs* male)	1.0 (0.7, 1.5), *P* = 0.9	1.3 (0.8, 2.0), *P* = 0.3
Age (years)	1.0 (0.9, 1.1), *P* = 1.0	1.1 (1.0, 1.2), *P* = 0.02*
Disease duration (years)	1.0 (0.9, 1.1), *P* = 0.9	1.0 (0.9, 1.1), *P* = 0.6
ILAR class		
Oligoarticular persistent	0.9 (0.5, 1.5), *P* = 0.7	1.2 (0.6, 2.1), *P* = 0.6
Oligoarticular extended	0.7 (0.4, 1.2), *P* = 0.2	0.9 (0.5, 1.8), *P* = 0.8
Polyarticular RF negative	[base]	[base]
Polyarticular RF positive	0.4 (0.2, 0.9), *P* = 0.02*	0.3 (0.1, 0.7), *P* = 0.009*
Systemic	0.9 (0.4, 2.3), *P* = 0.9	0.5 (0.1, 1.6), *P* = 0.2
Psoriatic	0.8 (0.4, 1.7), *P* = 0.6	0.9 (0.4, 1.9), *P* = 0.8
Enthesitis related	0.8 (0.4, 1.8), *P* = 0.7	1.0 (0.5, 2.3), *P* = 1.0
Undifferentiated	0.5 (0.1, 1.8), *P* = 0.3	0.3 (0.1, 1.7), *P* = 0.2
Steroids use (*vs* no use)	1.1 (0.7, 1.8), *P* = 0.5	1.0 (0.6, 1.7), *P* = 0.9
Active joint count (per joint)	1.0 (1.0, 1.0), *P* = 0.7	1.0 (1.0, 1.1), *P* = 0.2
PGA of disease activity (per cm)	0.9 (0.8, 1.0), *P* = 0.03*	0.9 (0.8, 1.0), *P* = 0.06
Parent/patient global assessment of well-being (per cm)	1.0 (0.9, 1.1), *P* = 0.6	1.0 (0.9, 1.2), *P* = 0.7
Pain VAS (per cm)	1.0 (0.9, 1.1), *P* = 0.9	1.0 (0.9, 1.1), *P* = 0.7
CHAQ (per unit)	1.0 (0.7, 1.5), *P* = 1.0	1.1 (0.7, 1.6), *P* = 0.8
ESR (mm/h)	1.0 (1.0, 1.0), *P* = 0.2	1.0 (1.0, 1.0), *P* = 0.3
MTX dose (mg/m^2^)		
≤7.5	[base]	[base]
>7.5–≤10	0.7 (0.4, 1.2), *P* = 0.2	1.5 (0.7, 3.2), *P* = 0.3
>10–≤12.5	0.8 (0.4, 1.7), *P* = 0.5	1.7 (0.7, 4.2), *P* = 0.3
15	0.8 (0.4, 1.8), *P* = 0.6	1.4 (0.5, 3.5), *P* = 0.5
≥17.5	1.2 (0.5, 2.8), *P* = 0.7	1.6 (0.6, 4.3), *P* = 0.4

Using imputed data. *P < 0.05.

VAS: visual analogue scale.

## Discussion

This is one of the largest prospective observational studies to describe treatment survival and ADR occurrence in children receiving MTX for JIA. More than half were no longer receiving MTX as their sole DMARD treatment within 2 years of starting treatment, although 8% stopped for disease remission. Fifty-two per cent added a biologic to their MTX following ineffectiveness. Overall, 37% of patients experienced an ADR during the first 2 years of MTX monotherapy, the majority of which were gastrointestinal or elevated liver enzymes. The occurrence of a gastrointestinal ADR was less likely in patients with polyarticular RF-positive JIA, but predicted by older age at the start of MTX treatment.

MTX is the recommended first-line DMARD for children with JIA. However, by 1 year only 65% of patients remained on MTX as monotherapy. Other cohort studies looking at patients prior to 2012 have found higher proportions of children remaining on MTX as the sole therapy (78–93% at 1 year) [6, 13], although these older studies reported few patients starting a biologic, with one excluding biologic patients completely. With more than half of the children in the current study reported to start a biologic therapy in combination with MTX, this suggests a more aggressive approach to JIA treatment in recent years [20].

Patients with a higher PGA at the start of MTX therapy, indicating those with more severe disease, were less likely to experience an ADR. It is possible that these patients may be more likely to tolerate side effects in the setting of more severe disease, balancing the positive effects of therapy with the negative. A previous study found that patients with polyarticular JIA had five times the odds of MTX intolerance compared with oligoarticular [6]. The current analysis found that patients with polyarticular RF-positive JIA had reduced odds of an ADR compared with those with polyarticular RF-negative JIA. It is possible that the previous study did not have enough power to detect the difference between RF status in polyarticular patients. In a UK survey of adolescents and adults with inflammatory arthritis taking MTX, adolescents had six times the odds of experiencing nausea compared with adults [8]. The current analysis found that for every year increase in age of patients starting MTX, there was a 10% increase in the odds of a gastrointestinal ADR, including nausea. This may support the evidence that adolescents are more likely to experience or report nausea with MTX compared with younger children.

As is typical of observational data, there were missing data and not all patients (*n* = 62) had the full 2 years of follow-up (due to patient relocation, change in hospital or delays in centres reporting to the study), although the statistical methods used in this analysis were able to account for this. The ADRs included reflected the most common ADRs associated with MTX. The events included in this study were reported to the study by the clinician and not directly by the patient, which may have resulted in some ADRs being missed or not reported to the register. Similarly, mild events may not have been recorded by the physician and therefore were not captured by the study, thus the estimates presented here may be an underestimate of the true burden of taking MTX. Although reports of elevated liver enzymes were common, the study did not apply a specific cut-off level for these or capture actual enzyme levels. Finally, the route of administration was unavailable for the majority of patients, thus this could not be investigated, and the timing of nausea and vomiting in relation to the MTX dose was not recorded. Therefore it was unknown whether these events were following MTX therapy or in anticipation of MTX. While events deemed not associated with MTX were excluded, it is possible that some events included were not related to MTX therapy.

This study has highlighted that after 2 years, approximately half of the patients remained on MTX monotherapy, with 8% of those who stopped MTX doing so for disease remission. Moreover, only one-third of the patients experienced an ADR within the first 2 years of therapy. This supports the opinion that MTX is considered an effective and safe first-line DMARD treatment for patients with JIA. However, further studies are needed to investigate the potential for concomitant therapy or support for the small proportion of children likely to experience an ADR, particularly as initial prevention of symptoms such as nausea is likely the best approach. In addition, as clinical factors alone cannot fully predict the occurrence of ADRs, further biological studies, including genetic markers, should be undertaken to continue to search for predictors of adverse MTX drug effects.

## Supplementary Material

kez048_Supplementary_Data
