# Supplementary material for: Methotrexate persistence and adverse drug reactions in patients with juvenile idiopathic arthritis
Source: Rheumatology (Oxford). 2019 Mar 8;58(8):1453–8. doi: 10.1093/rheumatology/kez048 (PMC6649753; doi:10.1093/rheumatology/kez048)
Supplement: kez048_Supplementary_Data [file kez048_supplementary_data.docx]

**SUPPLEMENTARY MATERIAL**

**Supplementary Table S1: Baseline characteristics of 577 patients with JIA registered at the start of methotrexate monotherapy.**

|  | **All JIA Patients** |
| --- | --- |
| **Gender, female** [N=577] | 393 (68%) |
| **Age at registration, years** [N=577] | 9 (4, 13) |
| **Disease duration, years** [N=569] | 0 (0, 1) |
| **ILAR Category** [N=577] | - |
| Systemic arthritis | 27 (5%) |
| Oligoarthritis: persistent | 123 (21%) |
| Oligoarthritis: extended | 85 (15%) |
| Polyarthritis RF negative | 188 (33%) |
| Polyarthritis RF positive | 49 (8%) |
| Psoriatic arthritis | 46 (8%) |
| Enthesitis related arthritis | 45 (8%) |
| Undifferentiated | 14 (2%) |
| **History of chronic anterior uveitis** [N=540*] | 51 (9%) |
| **Concomitant steroids** [N=577] | 151 (26%) |
| **Methotrexate dose (mg/m^2^)** [N=577] | 15 (10, 17.5) |
| ≤7.5mg/m^2^ | 99 (17%) |
| 7.5<x≤10mg/m^2^ | 117 (20%) |
| 10<x≤12.5mg/m^2^ | 61 (11%) |
| 15mg/m^2^ | 144 (25%) |
| 17.5mg/m^2^ | 31 (5%) |
| >17.5mg/m^2^ | 125 (22%) |
| **Disease Activity** | - |
| Active Joint Count (71 joints) [N=532*] | 4 (2, 8) |
| Limited Joint Count (71 joints) [N=517*] | 3 (1, 5) |
| Physician global assessment of disease activity(10cm VAS) [N=384*] | 3 (2, 5) |
| Parent (patient) global assessment of wellbeing (10cm VAS) [N=406*] | 4 (1, 6) |
| Pain VAS (10cm) [N=394*] | 4 (2, 7) |
| Childhood Health Assessment Questionnaire (CHAQ, 0-3*) [N=406*] | 0.9 (0.3, 1.5) |
| Erythrocyte sedimentation rate (ESR, mm/hr) [N=499*] | 14 (7, 30) |
| C-reactive protein (CRP, mg/l) [N=506*] | 5 (4, 12) |
| JADAS-71 [N=278*] | 12 (7, 21) |

Results presented at median (interquartile range) or N (%). *More than 5% missing data. ILAR: International League of Associations for Rheumatology; VAS: visual analogue scale; JADAS-71: joint juvenile arthritis disease activity score; CHAQ: Childhood Health Assessment Questionnaire

**Supplementary Table S2: Stop reasons of 310 (54%) patients who stopped methotrexate monotherapy within the first two years.**

|  | **Patients** |
| --- | --- |
| **N** | **310** |
| **Remission** | **24 (8%)** |
| **Ineffectiveness** | **185 (60%)** |
| Stopped methotrexate | 24 (8%) |
| Started biologic | 161 (52%) |
| **Adverse Event** | **78 (25%)** |
| **Patient / Family Decision^#^** | **10 (3%)** |
| **Unknown** | **13 (4%)** |

Data presented as number (percentage). ^#^Majority were described as injection stress or unable to tolerate without further details.

**Supplementary Table S3: Occurrence of Adverse Drug Reactions in 212 (37%) of the 577 JIA patients.**

|  | **All Patients**  **N=577** | **Proportion of all patients** | **Proportion experiencing an ADR^#^** | **Proportion resulting in permanent discontinuation^#^** |
| --- | --- | --- | --- | --- |
| **Any ADR** | 212 | 37% | 100% | 34 (16%) |
| Gastrointestinal (including nausea, vomiting) | 145 | 25% | 68% | 21 (14%) |
| Raised liver enzymes | 56 | 10% | 26% | 5 (9%) |
| Rash | 22 | 4% | 10% | 0 |
| Psychological symptoms (including anxiety, needle phobia) | 6 | 1% | 3% | 1 (17%) |
| Drug hypersensitivity (including injection site reaction) | 5 | 0.9% | 2% | 0 |
| Leukopenia | 4 | 0.7% | 2% | 0 |
| Other* | 11 | 2% | 5% | 7 (64%) |

Data presented as number (percentage). ADR = adverse drug reaction. ^#^Percentage of children who experienced the ADR. *Others include: alopecia, cough, dizziness, drug intolerance (x3) headache (x3), infection (x2). ADR: Adverse Drug Reactions
